# Social and geographic inequalities in water, sanitation and hygiene access in 21 refugee camps and settlements in Bangladesh, Kenya, Uganda, South Sudan, and Zimbabwe

**DOI:** 10.1186/s12939-022-01626-3

**Published:** 2022-02-19

**Authors:** Alhelí Calderón-Villarreal, Ryan Schweitzer, Georgia Kayser

**Affiliations:** 1 Department of Family Medicine and Public Health, University of California, San Diego (UCSD), San Diego, California USA; 2 School of Public Health, San Diego State University (SDSU), San Diego, California USA; 3 Former United Nations Refugee Agency (UNHCR) WASH Officer, Geneva, Switzerland; 4 Division of Global Health, Herbert Wertheim School of Public Health and Human Longevity, UCSD, San Diego, California USA

**Keywords:** WASH, Refugees, Women, Menstrual health, WASH access index, Asia, Africa

## Abstract

**Introduction:**

Many refugees face challenges accessing water, sanitation, and hygiene (WASH) services. However, there is limited literature on WASH access for refugee populations, including for menstrual health services. Unmet WASH access needs may therefore be hidden, amplifying morbidity and mortality risks for already vulnerable refugee populations. The aim of this study was therefore to quantitatively analyze WASH access among refugee camps, with a focus on households with women of reproductive age.

**Methods:**

This was a cross-sectional study that utilized the Standardized WASH Knowledge, Attitude and Practice (KAP) Survey. A total of 5632 household questionnaires were completed by the United Nations Refugee Agency in 2019 in 21 refugee camps and settlements in Bangladesh, Kenya, South Sudan, Uganda, and Zimbabwe. WASH access (14 items) and social and geographic stratifiers were analyzed at the household-level including the refugee camp, country of the settlement, having women of reproductive age, members with disability/elderly status, and household size. We calculated frequencies, odds ratios, and performed bivariate and multivariate analyses to measure inequalities. We developed a Female WASH Access Index to characterize WASH access for households with women of reproductive age.

**Results:**

Most refugee households had high levels of access to improved water (95%), low levels of access to waste disposal facility (64%) and sanitation privacy (63%), and very low access to basic sanitation (30%) and hand hygiene facility (24%). 76% of households with women of reproductive age had access to menstrual health materials. WASH access indicators and the Female WASH Access Index showed large inequalities across social and geographic stratifiers. Households with disabled or elderly members, and fewer members had poorer WASH access. Households with women of reproductive age had lower access to basic sanitation.

**Conclusions:**

Large inequalities in WASH access indicators were identified between refugee sites and across countries, in all metrics. We found high levels of access to improved water across most of the refugee camps and settlements studied. Access to basic hygiene and sanitation, sanitation privacy, waste disposal, and menstrual health materials, could be improved across refugee sites. Households with women of reproductive age, with 4+ members, and without members with disability/elderly status were associated with higher WASH access. The female WASH access index piloted here could be a useful tool to quickly summarize WASH access in households with women of reproductive age.

**Supplementary Information:**

The online version contains supplementary material available at 10.1186/s12939-022-01626-3.

## Highlights

Most camps have improved sanitation, albeit often shared and with poor privacy.

Most households had access to soap, but few had dedicated hand hygiene facilities.

Households with women of reproductive age had lower access to basic sanitation.

The Female WASH Access Index summarizes and compares female access across camps.

Households with disabled or elderly members, and fewer members had poorer WASH access.

**Supplementary Information:**

The online version contains supplementary material available at 10.1186/s12939-022-01626-3.

## Introduction

At the end of 2020, approximately 82.4 million people were forcibly displaced due to persecution, conflict, violence or human rights violations [1]. Of these; 26.4 million were refugees who are “unable or unwilling to return to their country of origin owing to a well-founded fear of being persecuted for reasons of race, religion, nationality, membership of a particular social group, or political opinion” [2]. Many refugees face challenges accessing basic services including water, sanitation and hygiene (WASH) services [3–7]^(p)^, which increases their risk of mortality and morbidity [4, 8]. Estimates of disease burden highlight high levels of poor health and associated social burden among refugee populations, which are highly related to inadequate WASH facilities and lack of access to clean water [9, 10]. Prevalence of diarrheal disease in refugee camps are often significantly higher than their host countries [9]. Handwashing with soap and water has been shown to reduce the risk of diarrheal disease by 23 to 40% [11, 12]. Likewise, inconsistent soap availability for hand washing has been shown to be commonplace in refugee settlements and camps in Uganda and South Sudan [6, 13].

Within the refugee population, women and girls have unique vulnerabilities related to inadequate WASH access. The responsibility for water collection—which in rural areas of developing countries often involves travelling far distances—falls more heavily on women and girls [14, 15]. Despite often having the primary responsibilities within the household for managing WASH services, women and girls often lack decision making control over them [15–17]. This can also reduce time available for education or economic activities often referred to as “time poverty” [15, 17, 18].

Women of reproductive age, (from 15 to 49 years-of age) have an additional potential vulnerability as they must manage menstrual periods, and therefore have specific WASH needs [19]. In many social and cultural contexts menstruation is associated with stress, shame and embarrassment, and with taboos that negatively impact and reinforce gender inequities and social exclusion [20, 21]. Every month, women and girls require access to menstrual materials to manage bleeding, private facilities to change menstrual materials, bathing facilities, clean water, toilet paper and/or soap and water to wash and dry themselves, and soak, wash, dry and/or dispose of used materials [19–22]. Without access to the necessary supplies and services for managing menstruation, women are at a greater risk for diseases, including urinary tract infections and toxic shock syndrome [23]. Female refugees face significant barriers; dignified, private and safe menstrual management is difficult, as they often have to share sanitation or hygiene facilities with males, multiple households, or with strangers [20, 24]. This has been associated with sexual harassment and gender based violence (GBV) in refugee camps and settlements) [18, 25, 26]. Research has shown that these factors can lead to decreased educational attendance and achievement and decreased employment and economic potential for women [24]. However, more evidence regarding access to menstrual health services for women in refugee camps and settlements is needed [27].

There are other factors that might contribute to WASH vulnerabilities either at the individual or household level. One factor is household size. Research in urban slums in Kenya found a positive association between household size and water consumption [28], washing hands, and sanitation access [29]. However, there are limited published studies looking at the relationship between household size and WASH access in refugee camps and settlements [30].

Disability is another factor that can lead to vulnerability. The World Health Organization (WHO) estimates that 15% of the world’s population is living with some form of disability, and an additional 3.8% of people age 15 years or older have functional limitations, that often require healthcare services [31]. War, natural disasters, and other forms of human conflicts that displace people can also lead to physical disabilities or other psycho-social disorders. The unique needs of refugees living with disabilities may be overlooked during acute humanitarian crises, causing them to experience multiple disadvantages [32]. Currently, there is insufficient data on the prevalence of disabilities within the global refugee population, which hinders a better understanding of the risks they face in displacement [33]. One study conducted in Syria found that 37% of the total population above 12 years of age has a disability suggesting that the prevalence and negative impacts of living with a disability are more pervasive in crisis-affected countries than in the general population [33]. One of the WHO recommendations for increasing inclusion and equity for people with disabilities involves assuring their access to WASH [31]. Some research, however, demonstrates being disabled or elderly may reduce access to WASH services [34]. A disability can increase out-of-pocket health expenditures or reduce an individual’s earning potential, thus impacting their ability to pay for services. Additionally, the disabled or elderly may not be physically capable to carry water or walk the distance required to access an improved main water source, especially in households with few members [35]. Finally, the disabled or elderly may find it difficult to use sanitation or hygiene facilities that have not been designed with their specific needs in mind.

The identification of gaps in equity of WASH access is an important first step in improving the health and wellbeing of those living in refugee camps and settlements. Closing these gaps has become a main focus of the Sustainable Development Goals (SDGs) including Goal 6, which proposes to “ensure availability and sustainable management of water and sanitation for all”, and goals 3 and 5 related with health and gender equity [36, 37]. Typically, monitoring WASH access and quality of WASH services involves measurement of data for determination of several indicators concurrently. For example, the United Nations Refugee Agency (UNHCR) WASH Monitoring System includes a total of 40 key indicators for monitoring WASH services at the household, community, and institutional levels (i.e., schools and health care facilities). It can be difficult to interpret multiple indicators concurrently when trying to assess WASH access for a given location. Indices, as aggregated indicators, are increasingly recognized as powerful tools to support decision-makers who need reliable data on which to base strategic planning, and target and prioritize funding [38, 39]. Indices have been developed for measuring WASH poverty in rural Kenya [39], the sustainability of sanitation services in South Korea and Argentina [40], and for evaluating the human right to water [41], and general hygiene practices [42]. One notable analysis used a WASH index to facilitate WASH related assessments in Greek refugee camps, to provide necessary information for efficient program planning and implementation of humanitarian interventions [43]. We are unaware of a WASH index that specifically seeks to quantify female needs in refugee settings.

The aim of this study was to quantitatively analyze WASH access in refugee camps across countries in Africa and Asia with a focus on households with women of reproductive age and vulnerable populations. These data were used to construct a female WASH access index. Drawing on the resulting WASH index, we compare social and geographic stratifiers among refugee camps and settlements in Kenya, Uganda, South Sudan, Bangladesh, and Zimbabwe, using data collected by UNHCR in 2019 [44, 45]. This research could be used to strategize allocation of scarce resources towards interventions that improve the health, wellbeing, and dignity of vulnerable groups, including women, people with disabilities and the elderly in refugee camps and settlements.

## Methods

### Study design and data collection

We conducted a cross-sectional study of access to WASH services in refugee camps and settlements using data from WASH Knowledge, Attitude and Practice (KAP) surveys. WASH KAP surveys were carried out by UNHCR and its partners in 2019 in 21 refugee camps and settlements in Bangladesh [12], Kenya [2], South Sudan [2], Uganda [2], and Zimbabwe [1]. WASH KAP survey questionnaires were completed in 21 refugee camps and settlements among 5632 randomly selected households, representing a total of 279,092 households (1,562,914 refugees). On average, 2% of the households were sampled across the refugee sites (range from 1.1 to 23%). Sample size of households per refugee camp ranged from *n* = 102 (Camp 4 Extension, Bangladesh) to *n* = 837 (Kakuma, Kenya). Specific details on the probabilistic sampling approach used in each of the camps can be consulted in the meta data available at UNHCR Microdata Library [44]. A household is defined as “a group of people who live together and routinely eat out of the same pot” [46]. The total population represented in each camp was obtained from WASH KAP 2019 refugee camp/country report or the UNHCR public data for each camp for the same year. All camps and settlement populations are described in Table 1 [47–50].

Fourteen specific WASH indicators were included in the analysis: use of an improved water source, access to greater than 15 L/p/d of improved/protected water, access to greater than 20 L/p/d of improved/protected water, practicing open defecation, accessing unimproved sanitation, accessing limited sanitation, accessing basic sanitation, having sanitation privacy, presenting soap within 1-min, accessing basic hand hygiene facility, having toilet paper/water in sanitation facility, having a bathing facility, accessing menstrual health materials in the last period, and having a solid waste disposal facility. Social and geographic stratifiers included: location (e.g., country, refugee camp or settlement), household size, having at least one woman of reproductive age (defined as 15 to 49 years old), and having at least one member with disability and/or elderly (60+) person in the household. Selected questions from the KAP can be read in the Supplemental Questionnaire. Only refugee households were included in the analysis and a small number of non-refugee households (i.e., host community households were excluded from the analyses.

### WASH indicators definitions

WASH indicators were dichotomized (access/no access) using definitions used by UNHCR and definitions used by the Joint Monitoring Programme (JMP) of the United Nations International Children’s Emergency Fund (UNICEF) and the WHO.

Drinking water was ‘improved’ or ‘unimproved.’ Improved main water source were “those that have the potential to deliver safe water by nature of their design and construction”, and included: piped water (public taps/standpipes, piped connections to a house or neighbor’s house), boreholes or tubewells, protected dug well, protected springs, rainwater, packaged (bottled or sachets), and delivered water (water sellers/kiosks or tanker trucks) [11]. Unimproved or surface water sources included unprotected dug wells, unprotected springs, surface water (lake, pond, dam, and river), ‘other’ and ‘don’t know’. Average number of liters per person per day (L/p/d) of potable protected water were classified in two indicators, ≥15 L/p/d (UNHCR and SPHERE emergency target) and ≥ 20 L/p/d (UNHCR post emergency target) [20].

Sanitation was classified following JMP definitions into the following categories: basic, limited, improved, unimproved and open defecation. Improved sanitation facilities were those designed to hygienically separate excreta from human contact and included: household and communal latrines with plastic or concrete slabs [11]. Basic sanitation was defined as the use of improved facilities which were not shared with other households [11]. Limited sanitation was defined as the use of improve facilities shared between two or more households with a plastic or concreate slab, usually these were communal latrines [11]. Unimproved sanitation was defined as all latrines without a plastic or concreate slab or bucket toilets [11]. Open defecation was defined as a household that that admitted to defecating in the open or in plastic bag or which stated that they did not have access to any facility. A sanitation facility provided “privacy” if it had a door that closed and had a lock.

Handwashing facilities were “fixed or mobile and include a sink with tap water, buckets with taps, tippy-taps, and jugs or basins designated for handwashing” [11]. Basic hygiene was “the availability of a handwashing facility on premises with soap and water”, these could be a household facility and/or a handwashing station in the sanitation facility [11]. Bathing facilities were defined as present if the household had a designated or nearby bathing facility observed by the interviewer, and not present if they did not have such a facility. Soap included “bar soap, liquid soap, powder detergent, and soapy water” [11] and access was considered if a household member was able to present any of the aforementioned items to the interviewer within one minute [20].

Access to menstrual health services meant access to toilet paper or water for cleaning, solid waste disposal options, and menstrual health products (e.g., disposable pads, reusable pads, reusable clothe, tampons, cotton, and menstrual cups). Response options that weren’t considered adequate menstrual health products included: layers of underwear, nothing and other (unspecified) [45]. Toilet paper or cleaning water were accessible if they were available in the physical spaces where women change their menstrual health products. An adequate solid waste disposal facility was defined as a household pit, communal pit, household bin or street bin/container for garbage collection. Inadequate solid waste disposal options included: an undesignated open area, designated open area, burying, burning, throwing it into a drain nearby, throwing it outside of the house/shed for animals to eat, and ‘other’ (unspecified).

### Female WASH access index

A Female WASH Access Index was created by combining numerous indicators specific to female WASH needs, using principal component analysis (PCA) [51]. The Female WASH Access Index included six WASH items: 1) main water source, 2) sanitation facility, 3) bathing facility, 4) access to menstrual health materials, 5) basic hygiene facility, and 6) solid waste disposal facility. The PCA score was transformed to a 0 to 1 index using a percentile function, where 0 represents the worst observed value and 1 represents the best. Index results are described as points.

### Stratifiers

To assess the inequality in access to WASH services, social and geographic stratifiers were identified. Twenty-one refugee camps and settlements were included: Tongogara Camp in Zimbabwe, Kyangwali and Palabek settlements in Uganda, Kakuma and Kalobeyei camps in Kenya, Pamir and Ajoung Thok camp in South Sudan, and Camps 1E, 1 W, 2E, 2 W, 3, 4, 4 Extension, 5, 17, 26, 27, Kutupalong and Nayapara Registered Camps (RC) in Bangladesh. Having at least one woman of reproductive age and having at least one person with a disability or who are elderly were dichotomous variables. Household size was an ordinal variable classified according to the number of members per household, categorized into three groups: 1–3, 4–6, and 7+ members.

### Statistical analysis

All calculations were adjusted using the survey weights provided with each database. However, as this analysis used data from multiple countries and surveys, all survey weights were standardized. This involved rescaling them to represent the entire population in each refugee camp. For the households with missing information regarding the sanitation facilities type of slab material (23%), we probabilistically imputed the value based on the proportions in the non-missing data. Missing values per indicator are described in Supplementary Table 1.

WASH access indicators and stratifiers were described for the total population and analyzed by refugee camp/settlement, household size, and presence of members with a disability or who are elderly.

We calculated frequencies, bivariate and adjusted multivariate odds ratios (OR and aOR respectively) and performed simple and multivariate analyses to identify WASH access inequities using stratifiers. Simple and multivariate logistic regression models were run for the 14 WASH access indicators (dependent variables) to test for associations with stratifiers (independent variables). A linear regression model was run using the Female WASH Index as the outcome, to assess for associations. The Kyangwali Settlement, Uganda was excluded from the regression analysis because no data were available for one of the covariates—disability/elderly status.

Significant statistical values included confidence intervals at 95% and *p* values of < 0.05. All analyses were conducted using R Version 4.0.2.

## Results

The average household size was 5.6 members. Most of the households (86%) had at least one woman of reproductive age. Almost a quarter of the households had at least one person with a disability or who were elderly (25%). Table 1 has additional information on household characteristics by site and country.

**Table 1:** Household characteristics in 21 refugee camps in 2019

Refugee camp	Sampled households	Population represented^**a**^	Household represented	Members per household (mean)	Households with at least one woman at reproductive age (sampled, represented, %)			Households with member/s with disability or who are elderly (sampled, represented, %)		
Total	5632	1,562,914	279,092	5.6	4860	240,856	86.3	1385	68,657	24.6
Refugee camp										
Bangladesh	1683	349,394	68,509	5.1	1548	63,028	92.0	370	15,072	22.0
Camp 17	123	16,336	3203	5.1	113	2944	91.9	25	650	20.3
Camp 1E	140	37,782	7408	5.1	127	6719	90.7	31	1637	22.1
Camp 1 W	130	37,996	7450	5.1	115	6593	88.5	33	1892	25.4
Camp 21	120	16,468	3830	4.3	103	3286	85.8	19	605	15.8
Camp 26	118	40,627	7387	5.5	116	7261	98.3	29	1817	24.6
Camp 27	114	14,914	2983	5.0	108	2825	94.7	34	889	29.8
Camp 2E	121	25,657	5702	4.5	117	5513	96.7	23	1083	19.0
Camp 2 W	118	23,586	4914	4.8	110	4580	93.2	27	1125	22.9
Camp 3	119	35,598	7416	4.8	103	6422	86.6	26	1617	21.8
Camp 4	117	29,859	5855	5.1	108	5404	92.3	17	849	14.5
Camp 4 Ext	102	6836	1486	4.6	93	1355	91.2	23	334	22.5
Camp 5	118	24,437	5199	4.7	105	4627	89.0	15	660	12.7
Kutupalong	126	16,713	2653	6.3	112	2358	88.9	31	653	24.6
Nayapara	117	22,585	3475	6.5	115	3416	98.3	38	1129	32.5
Kenya	981	189,692	29,183	6.5	766	22,792	78.1	180	5341	18.3
Kakuma	837	153,593	23,272	6.6	640	17,803	76.5	151	4212	18.1
Kalobeyei	144	36,099	6017	6.0	122	5090	84.6	28	1161	19.3
South Sudan	1466	73,406	11,652	6.3	1363	10,836	93.0	397	3158	27.1
Ajoung Thok	727	39,309	6142	6.4	680	5743	93.5	201	1695	27.6
Pamir	739	34,097	5590	6.1	683	5165	92.4	196	1481	26.5
Uganda	849	358,131	67,572	5.3	711	56,625	83.8	288	22,907	33.9
Kyangwali	403	114,716	22,943	5.0	349	19,846	86.5	NA	NA	NA
Palabek	446	214,477	38,996	5.5	367	32,094	82.3	151	13,220	33.9
Zimbabwe	653	14,469	2837	5.1	515	2238	78.9	107	465	16.4
Tongogara	653	14,469	2837	5.1	515	2238	78.9	107	465	16.4

Source: Table created from the Standardized Water, Sanitation and Hygiene, Knowledge, Attitude and Practice Surveys, UNHCR, 2019

^**a**^Population represented were obtained from UNHCR WASH Indicators Summarized by Location and KAP WASH UNHCR Survey Reports

### WASH access

**Fig. 1:**
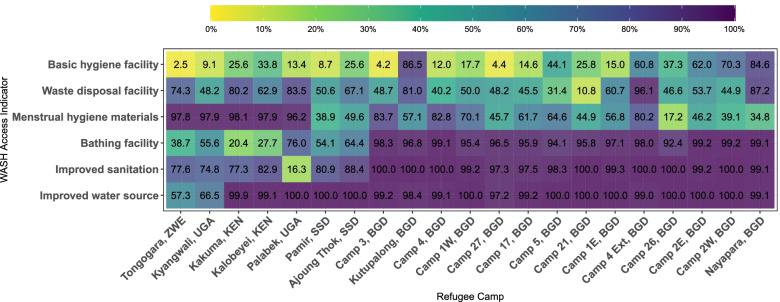
Percentage of Household with WASH Access Indicators in 21 Refugee Camps in 2019. This heat map illustrates the percentage of households with WASH access among 21 refugee camps in Bangladesh, Kenya, South Sudan, Uganda and Zimbabwe in 2019. On the y axis six selected WASH indicators are shown, and on the x axis the name of refugee camps followed by the abbreviation of the settlement country. The lighter the color in the box, the lower the percentage of households with WASH access in the corresponding refugee camp. This figure was created from the Standardized WASH KAP Surveys, UNHCR, 2019

Figure 1 provides a summary of WASH access across sites and selected WASH indicators included in the female WASH index. Supplementary Fig. 1 also provides WASH access indicators by country. Table 2 has a summary of WASH indicators and the female WASH access index by country, site and stratifier.

**Table 2:** WASH access indicators per social and goegraphic stratifiers in 21 refugee camps in 2019

Social/geographic stratifier	Water			Sanitation						Hygiene						Female WASH index°
	Improved water source (%)	≥15 L/p/d of improved/protected water (%)	≥20 L/p/d of improved/protected water (%)	Open defecation (%)	Unimproved sanitation (%)	Improved sanitation (%)	Limited saniatation (%)	Basic sanitation (%)	Sanitation privacy (%)	Soap presented within 1 min (%)^a^	Basic hand hygiene facility(%)^b^	Toilet paper/water in sanitation facility (%)^c^	Bathing facility (%)^a^	Menstrual health materials in the last period (%)^a,c^	Solid waste disposal facility (%)^a^	
Total	95.1	50.6	36.9	4.6	23.2	72.2	42.7	29.5	62.6	80.0	23.5	62.6	68.6	76.1	64.1	0.50
Refugee camp																
Bangladesh	99.5	40.4	28.2	0.6	0.0	99.3	97.3	2.0	91.3	98.0	34.6	78.7	96.7	54.2	51.1	0.70
Camp 17	99.2	18.7	11.4	2.5	0.0	97.5	96.7	0.8	90.2	100	14.6	69.2	95.9	61.7	45.5	0.65
Camp 1E	100	43.6	26.4	0.7	0.0	99.3	97.9	1.4	97.9	97.7	15.0	65.6	97.1	56.8	60.7	0.66
Camp 1 W	100	33.8	21.5	0.8	0.0	99.2	99.2	0.0	94.6	96.9	17.7	86.9	95.4	70.1	50.0	0.63
Camp 21	100	42.5	27.5	0.0	0.0	100	98.3	1.7	93.3	96.6	25.8	71.4	95.8	44.9	10.8	0.68
Camp 26	100	86.4	76.3	0.0	0.0	100	91.5	8.5	78.8	99.1	37.3	96.6	92.4	17.2	46.6	0.82
Camp 27	97.4	15.8	7.9	2.7	0.0	97.3	92.0	5.4	86.0	98.0	4.4	63.8	96.5	45.7	48.2	0.61
Camp 2E	100	53.7	37.2	0.8	0.0	99.2	99.2	0.0	96.7	98.3	62.0	59.8	99.2	46.2	53.7	0.84
Camp 2 W	100	62.7	50.8	0.0	0.0	100	99.2	0.8	96.6	100	70.3	73.6	99.2	39.1	44.9	0.84
Camp 3	99.2	10.1	0.8	0.0	0.0	100	99.2	0.8	92.4	97.5	4.2	89.8	98.3	83.7	48.7	0.57
Camp 4	99.1	17.1	11.1	0.0	0.0	100	98.3	1.7	85.5	97.4	12.0	84.8	99.1	82.8	40.2	0.60
Camp 4 Ext	99.0	15.7	9.8	0.0	0.0	100	100	0.0	99.0	98.0	60.8	67.4	98.0	80.2	96.1	0.68
Camp 5	100	16.9	5.9	1.7	0.0	98.3	97.5	0.8	86.4	98.2	44.1	58.6	94.1	64.6	31.4	0.68
Kutupalong	98.4	34.9	19.0	0.0	0.8	99.2	99.2	0.0	97.6	95.9	86.5	83.1	96.8	57.1	81.0	0.56
Nayapara	99.1	70.1	50.4	0.9	0.0	99.1	96.6	2.6	93.2	98.3	84.6	98.3	99.1	34.8	87.2	0.89
Kenya	99.7	46.8	37.2	3.7	18.0	78.3	5.6	72.7	69.2	82.5	27.2	49.3	21.8	98.1	76.9	0.37
Kakuma	99.9	46.0	36.1	1.9	20.9	77.3	4.2	73.1	71.5	84.2	25.6	55.4	20.4	98.1	80.2	0.36
Kalobeyei	99.1	50.2	42.0	11.7	5.4	82.9	11.8	71.0	59.5	75.2	33.8	25.5	27.7	97.9	62.9	0.41
South Sudan	100	68.1	47.9	1.1	14.0	85.0	10.6	74.4	56.9	70.5	17.7	NA	59.6	44.7	59.5	0.58
Ajoung Thok	100	75.5	55.6	0.8	10.7	88.4	7.4	81.0	60.9	85.4	25.6	NA	64.4	49.6	67.1	0.61
Pamir	100	59.5	39.1	1.4	17.7	80.9	14.2	66.7	52.2	53.3	8.7	NA	54.1	38.9	50.6	0.54
Uganda	88.3	64.5	46.7	10.3	53.6	36.1	13.0	23.1	8.1	63.3	11.9	49.4	68.9	96.8	71.2	0.36
Kyangwali	66.5	NA	NA	8.3	16.9	74.8	12.5	62.2	NA	75.4	9.1	NA	55.6	97.9	48.2	0.33
Palabek	100	64.5	46.7	11.3	72.4	16.3	13.3	3.0	8.1	56.9	13.4	49.4	76.0	96.2	83.5	0.37
Zimbabwe	57.3	53.1	43.3	6.1	16.3	77.6	24.6	53.0	NA	52.8	2.5	57.1	38.7	97.8	74.3	0.23
Tongogara	57.3	53.1	43.3	6.1	16.3	77.6	24.6	53.0	NA	52.8	2.5	57.1	38.7	97.8	74.3	0.23
**Social/geographic stratifier**	**Improved water source (%)**^a^	**≥15 L/p/d of improved/protected water (%)**	**≥20 L/p/d of improved/protected water (%)**	**Open defecation (%)**	**Unimproved sanitation (%)**	**Improved sanitation (%)**^a^	**Limited saniatation (%)**	**Basic sanitation (%)**	**Sanitation privacy (%)**	**Soap presented within 1 min (%)**^a^	**Basic hand hygiene facility (%)**^b^	**Toilet paper/water in sanitation facility (%)**^bb^	**Bathing facility (%)**^a^	**Menstrual health materials in the last period (%)**^a**,**c^	**Solid waste disposal facility (%)**^a^	**Female WASH index**^a^
At least one woman at repr. Age																
No	93.3	61.8	49.4	5.2	25.6	69.2	33.1	36.1	55.3	71.5	13.4	NA	56.8	NA	66.0	NA
Yes	95.4	48.8	34.9	4.5	22.7	72.8	44.3	28.5	63.8	81.4	25.2	63.7	70.5	76.0	63.8	0.56
Household size																
1 to 3 members	94.1	66.7	58.6	3.7	22.5	73.8	50.2	23.5	60.9	78.0	18.4	61.5	71.4	72.3	60.7	0.41
4 to 6 members	95.2	50.2	35.5	5.0	22.3	72.6	46.4	26.2	64.1	80.6	25.7	64.2	69.1	75.8	63.7	0.53
7 or more members	96.0	40.6	24.7	4.7	24.9	70.4	33.1	37.3	61.7	80.7	24.1	64.2	65.9	77.8	67.2	0.52
Member/s with disability or who are elderly																
No	99.0	48.7	36.1	4.5	20.7	74.7	48.2	26.6	64.9	81.3	25.6	64.9	68.9	72.6	64.9	0.54
Yes	99.1	56.4	39.3	3.0	34.1	62.9	41.7	21.1	55.7	78.6	25.1	59.9	74.7	76.6	70.7	0.47

Source: Table created from the Standardized Water, Sanitation and Hygiene, Knowledge, Attitude and Practice Surveys, UNHCR, 2019

^a^Female WASH index includes improved water source, basic hand hygiene facility, bathing facility, menstrual health materials, improved sanitation facility, and solid waste disposal only in households with at least one woman at reproductive age

^b^Basic hand hygiene is having available a handwashing facility with water and soap

^c^Toilet paper or water in the sanitation facility, and menstrual hygiene materials included just the households with at least one women of reproductive age

#### Water supply

Out of 21 sites, 19 had 97% or more of households accessing improved water supplies with Tongogara (Zimbabwe) and Kyangwali (Uganda) having less, at 57 and 67% respectively. On average, 51% of households had access to ≥15 l/p/d and 37% of households had access to ≥20 L/p/d (Fig. 1 and Table 2).

#### Sanitation

Only 30% of households across camps had access to basic sanitation and many households shared sanitation with other households (43% had limited sanitation). Additionally, 5% of households practiced open defecation, and 23% had access to unimproved sanitation. There was considerable variation between sites in terms of sanitation access. Uganda refugee settlements had a higher proportion of unimproved services (54%), and open defecation (10%) compared to other countries (ranging from 0% in Bangladesh to 18% in Kenya, and 1% in Bangladesh to 10% in Uganda). South Sudan and Kenya refugee camps had the highest basic sanitation rates (74 and 73%), and Bangladesh had the highest percentage of the population relying on limited sanitation (97%) compared to other countries (ranging from 6% in Kenya to 25% in Zimbabwe) (Fig. 1 and Table 2).

Across all sites, 63% of households had access to sanitation facilities that provided privacy. Sanitation privacy was lowest in Kyangwali Settlement in Uganda (8%), while the rest of the camps/settlements reported at least half of the households with sufficient sanitation privacy (mean 80%, median 90%).

#### Hygiene

We found limited access to basic hygiene across all sites. On average, 24% of households had access to a basic hand hygiene facility with observed water and soap (ranging from 3% in Tongogara, Zimbabwe to 87% in Kutupalong RC in Bangladesh). Across all sites, 69% of households had access to bathing facilities, ranging from 20% in Kakuma, Kenya, to 99% in Camp 2E and 2 W in Bangladesh (Fig. 1 and Table 2).

Mixed results were found for menstrual health access. Of households with women of reproductive age, 76% had access to menstrual health materials and 63% had toilet paper or water in their sanitation facility. High levels of menstrual health material access were observed in Uganda, Kenya, and Zimbabwe (> 96%) in comparison to the other two countries refugee camps (ranging from 45% in South Sudan to 54% in Bangladesh). Nevertheless, the aforementioned three countries had < 60% access to toilet paper or water in their sanitation facility. In Bangladesh, although 92% of households had at least one woman, only half had access to these materials, and remarkably, Camp 26 in Bangladesh had less than 20% of households with access.

#### Solid waste

Across all sites, 64% of households had access to a solid waste disposal facility. Bangladesh had sites with both the lowest and highest coverage with 11% (Camp 21) to 96% (Camp 4 extension) (Fig. 1 and Table 2).

### WASH inequality

WASH access indicators and odds ratios by these stratifiers are presented in Table 3. The following sections review these results.

**Table 3:** Multivariate associations of WASH access indicators across social stratifiers in 21 refugee camps in 2019

Social stratifier	WASH indicator	OR	CI95%		aOR	CI95%		***p***-value
Having at least one woman of reproductive age	Improved water source	2.06	1.61	2.63	1.94	1.42	2.66	**0.00**
	≥15 L/p/d of improved/protected water	0.63	0.53	0.74	0.95	0.79	1.14	0.59
	≥20 L/p/d of improved/protected water	0.53	0.45	0.62	0.93	0.77	1.11	0.42
	Open defecation	0.57	0.39	0.84	0.38	0.24	0.60	**0.00**
	Unimproved sanitation	0.90	0.73	1.10	0.90	0.71	1.13	0.37
	Limited saniatation	1.35	1.14	1.59	1.84	1.53	2.22	**0.00**
	Basic sanitation	0.87	0.75	1.02	0.67	0.56	0.80	**0.00**
	Sanitation privacy	1.12	0.93	1.36	1.07	0.87	1.32	0.50
	Soap presented within 1 min	1.67	1.40	1.98	1.75	1.44	2.13	**0.00**
	Basic hand hygiene facility	1.95	1.57	2.43	1.98	1.56	2.51	**0.00**
	Toilet paper/water in sanitation facility	NA	NA	NA	NA	NA	NA	NA
	Bathing facility	1.98	1.69	2.31	2.50	2.09	2.99	**0.00**
	Menstrual health materials/last period	NA	NA	NA	NA	NA	NA	NA
	Solid waste disposal facility	1.01	0.86	1.18	1.00	0.84	1.20	0.98
Having 4 to 6 members	Improved water source	1.74	1.37	2.20	1.66	1.23	2.25	**0.00**
	≥15 L/p/d of improved/protected water	0.48	0.41	0.55	0.49	0.41	0.57	**0.00**
	≥20 L/p/d of improved/protected water	0.36	0.31	0.41	0.37	0.31	0.43	**0.00**
	Open defecation	1.00	0.67	1.50	1.51	0.92	2.47	0.11
	Unimproved sanitation	0.95	0.78	1.15	0.98	0.79	1.21	0.85
	Limited saniatation	0.90	0.78	1.03	0.77	0.66	0.90	**0.00**
	Basic sanitation	1.15	1.00	1.33	1.26	1.07	1.48	**0.01**
	Sanitation privacy	1.08	0.91	1.28	1.05	0.88	1.25	0.62
	Soap presented within 1 min	1.19	1.01	1.41	1.02	0.85	1.23	0.81
	Basic hand hygiene facility	1.40	1.18	1.67	1.18	0.98	1.41	0.08
	Toilet paper/water in sanitation facility	1.19	0.97	1.46	1.20	0.97	1.47	0.09
	Bathing facility	1.12	0.97	1.29	0.88	0.75	1.03	0.11
	Menstrual health materials/last period	1.08	0.90	1.29	1.05	0.87	1.26	0.63
	Solid waste disposal facility	1.17	1.01	1.34	1.20	1.03	1.41	**0.02**
Having 7 or more members	Improved water source	1.95	1.50	2.52	1.73	1.25	2.40	**0.00**
	≥15 L/p/d of improved/protected water	0.33	0.28	0.39	0.33	0.28	0.39	**0.00**
	≥20 L/p/d of improved/protected water	0.22	0.19	0.25	0.22	0.19	0.26	**0.00**
	Open defecation	0.86	0.56	1.33	1.36	0.81	2.30	0.25
	Unimproved sanitation	1.05	0.86	1.27	1.06	0.85	1.31	0.63
	Limited saniatation	0.47	0.40	0.55	0.38	0.33	0.45	**0.00**
	Basic sanitation	2.01	1.73	2.33	2.36	2.00	2.78	**0.00**
	Sanitation privacy	0.95	0.80	1.13	0.93	0.78	1.12	0.46
	Soap presented within 1 min	1.14	0.96	1.35	0.96	0.79	1.16	0.66
	Basic hand hygiene facility	1.22	1.02	1.46	1.01	0.83	1.22	0.92
	Toilet paper/water in sanitation facility	1.27	1.02	1.57	1.29	1.04	1.60	**0.02**
	Bathing facility	1.03	0.89	1.20	0.77	0.65	0.91	**0.00**
	Menstrual health materials/last period	1.05	0.87	1.27	1.06	0.87	1.28	0.58
	Solid waste disposal facility	1.30	1.12	1.51	1.32	1.12	1.56	**0.00**
Having disability or elderly members	Improved water source	1.47	1.08	2.01	1.62	1.18	2.22	**0.00**
	≥15 L/p/d of improved/protected water	1.27	1.12	1.45	1.31	1.15	1.49	**0.00**
	≥20 L/p/d of improved/protected water	1.20	1.05	1.36	1.24	1.08	1.42	**0.00**
	Open defecation	0.64	0.41	1.02	0.60	0.37	0.95	**0.03**
	Unimproved sanitation	1.76	1.50	2.07	1.74	1.47	2.04	**0.00**
	Limited saniatation	0.82	0.72	0.93	0.89	0.78	1.02	0.11
	Basic sanitation	0.90	0.79	1.02	0.83	0.73	0.95	**0.01**
	Sanitation privacy	0.73	0.64	0.84	0.74	0.64	0.86	**0.00**
	Soap presented within 1 min	0.95	0.82	1.11	1.01	0.86	1.18	0.94
	Basic hand hygiene facility	1.08	0.93	1.26	1.15	0.99	1.34	0.07
	Toilet paper/water in sanitation facility	0.95	0.79	1.14	0.92	0.76	1.11	0.37
	Bathing facility	1.30	1.14	1.49	1.44	1.25	1.65	**0.00**
	Menstrual health materials/last period	1.02	0.87	1.18	1.01	0.86	1.18	0.91
	Solid waste disposal facility	1.14	1.00	1.31	1.14	0.99	1.30	0.07

Source: Table created from the Standardized Water, Sanitation and Hygiene, Knowledge, Attitude and Practice Surveys, UNHCR, 2019

^a^Reference groups: Households without women of reproductive age, households with 1 to 3 members, and households without members with disability or who are elderly

#### Women of reproductive age

When we analyzed the data in households with at least one woman of reproductive age, we found higher access to hygiene services (aOR: soap 1.75, basic hand hygiene 1.98, and bathing facility 2.50, *p* = 0.00), and an improved water source (OR: 1.94, *p =* 0.00), compared to households without women of reproductive age. Nevertheless, we found households with at least one woman of reproductive age had less access to basic sanitation (OR: 0.67, *p =* 0.00), as compared to households without women of reproductive age. OR and aOR are presented in Table 3.

#### Household size

Household size was positively associated with several WASH access indicators. Households with 4 to 6 members and 7+ members had the highest access to improved water (aOR: 1.66 and 1.73, *p* = 0.00) and basic sanitation indicators (aOR: 1.26 and 2.36, *p =* 0.00), in comparison to households with fewer members. Households with 7+ members were more likely to have toilet paper or water in their sanitation facility (aOR: 1.29, *p* = 0.02) and a solid waste disposal facility (aOR: 1.32, *p =* 0.00), in comparison to those with 1 to 3 members. Differences in access to menstrual health materials and soap access were not statistically significant when stratified by household size. Furthermore, households with 4 or more members had less access to > 15 L/p/d and > 20 L/p/d of improved/protected water (aOR: 4–6 members 0.49, 0.37, and 7+ members 0.33, 0.22, *p* = 0.00) in comparison to households with fewer members (Table 3).

#### Disabled and elderly

Households that had at least one member with a disability or who are elderly had lower access to basic sanitation (21% vs 27%), and sanitation privacy (56% vs 65%) in comparison to households without them (aOR: 0.83, *p* = 0.01; 0.74, *p =* 0.00). Household with members with these characteristics were more likely to utilize unimproved sanitation facilities (34% vs 21%; aOR: 1.74, *p =* 0.00). However, those households were more likely to have access to a bathing facility (aOR: 1.44, *p =* 0.00) (Table 3).

**Fig. 2:**
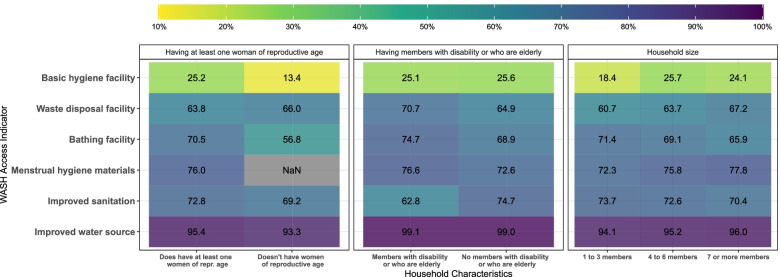
Percentage of Households with WASH Access by Household Characteristics in 21 Refugee Camps in 2019**.** This heatmap illustrates the percentage of households with WASH access according to having at least one woman at reproductive age (left), having members with disability or who are elderly (center), and household size (right) in 21 refugee camps in five countries in 2019. On the y axis, six selected WASH indicators are shown, and on the x axis the household characteristics. The lighter the color in the box, the lower the percentage of households with WASH access in the corresponding group. This figure was created from the Standardized WASH KAP Surveys, UNHCR, 2019

Figure 2 provides a visual representation of WASH indicators by household size, households with women of reproductive age, and households with members who are elderly or with a disability, to depict inequalities. Inequality gradients were evident across some of the WASH indicators, when compared across household size or members with a disability or who are elderly versus no such household member, especially among sanitation and hygiene indicators.

### Female WASH access index

**Fig. 3:**
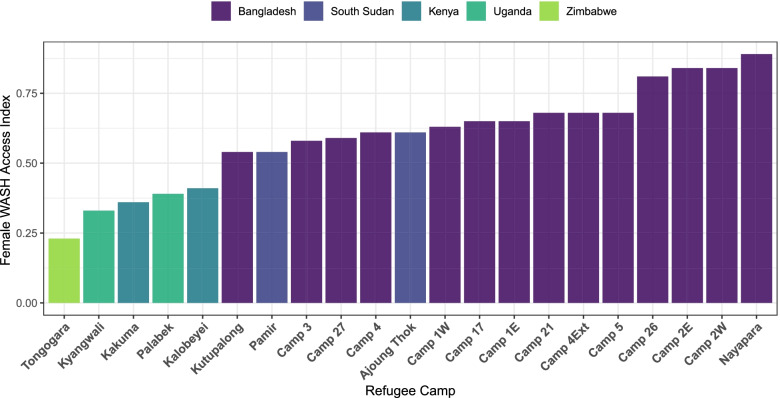
Female WASH Access Index in 21 Refugee Camps in 2019**.** This bar graph illustrates the Female WASH Access Index according to 21 refugee camps in five countries in 2019. On the y axis the Female WASH Access Index value is shown, and on the x axis the name of refugee camps. Each settlement country is indicated by a different color. This figure was created from the Standardized WASH KAP Surveys, UNHCR, 2019

**Fig. 4:**
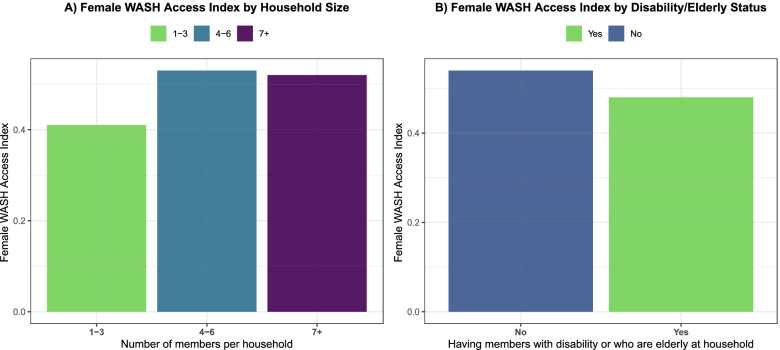
Female WASH Access Index by Household Characteristics in 21 Refugee Camps in 2019**.** This bar graph illustrates the Female WASH Access Index according to household size (left) and having members with disability or who are elder (right) in 21 refugee camps in five countries in 2019. On the y axis, is the Female WASH Access Index value, and on the x axis the corresponding household characteristic. This figure was created from the Standardized WASH KAP Surveys, UNHCR, 2019

The female WASH access index is described in Table 2 by social and geographic stratifiers. Tongogara, Zimbabwe had the lowest Female WASH Access Index score, corresponding with poorest values in terms of the WASH access indicators assessed, and Nayapara RC, Bangladesh had the highest score from all sites considered. In Fig. 3, the index is shown by site and in Fig. 4, by stratifier. The main tendencies observed in the WASH index were consistent with the patterns seen among the component indicators. Among households with women of reproductive age, households with fewer members, and with members with a disability or who are elderly, tended to have lower scores on the Female WASH Access Index.

**Fig. 5:**
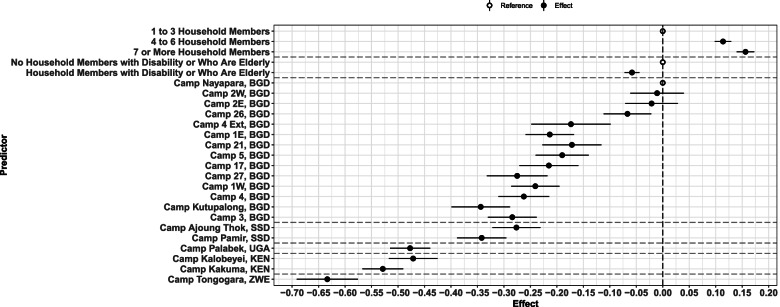
Multivariate Association with Female WASH Index Among 20 Refugee Camps Households in 2019. This graph illustrates the multivariate association with Female WASH Access Index among social and geographic stratifiers in 20 refugee camps households with at least one woman of reproductive age in 2019. On the y axis, are the social or geographic predictors, the name of each refugee camp is followed by the abbreviation of the settlement country. On the x axis the effect, outlined by the dotted line that represents the zero effect. Black points represent effects and white points references. The horizontal lines that cross each black point represents the CI95%. Kyangwali, Uganda refugee camp is not included in the multivariate logistic regression. This figure was created from the Standardized WASH KAP Surveys, UNHCR, 2019

Multivariate associations between stratifiers and the Index were described in Table 4 and Fig. 5. Having 4–6, and 7+ household members, were both associated with higher WASH access—as compared to 1–3 household members—with positive coefficients of 0.11 [CI95%: 0.10, 0.13] and 0.15 [CI95%: 0.14, 0.17] respectively. Having members with a disability or who are elderly in the household, was associated with a decrease in the Index with a coefficient of − 0.06 [CI95%: − 0.07, − 0.05]. Using the Nayapara, Bangladesh camp as a reference (as it had the highest WASH access), the Kutupalong camp in Bangladesh was associated with a decrease of − 0.32 [CI95%: − 0.38, − 0.27]. The Tongogara camp in Zimbabwe was associated with a decrease of − 0.64 [CI95%: − 0.69, − 0.58]. Most of the camp-level differences observed were statistically significant at the 95% confidence level.

**Table 4:** Multivariate Association with Female WASH Access Index among 19 refugee camps in 2019

Social/geographic stratifier	Coefficient	CI95%		p-value
Refugee camp				
Bangladesh				
Camp 17	−0.21	−0.27	−0.16	0.000
Camp 1E	−0.20	−0.25	−0.16	0.000
Camp 1 W	−0.24	−0.29	−0.20	0.000
Camp 21	−0.17	−0.23	−0.12	0.000
Camp 26	−0.05	−0.10	−0.01	0.017
Camp 27	−0.26	−0.31	−0.20	0.000
Camp 2E	−0.02	−0.07	0.03	0.364
Camp 2 W	−0.01	−0.06	0.04	0.636
Camp 3	−0.29	−0.33	−0.24	0.000
Camp 4	−0.26	−0.31	−0.22	0.000
Camp 4 Ext	−0.18	−0.25	−0.10	0.000
Camp 5	−0.19	−0.24	−0.14	0.000
Kutupalong	−0.32	−0.38	−0.27	0.000
Nayapara	0.00	0.00	0.00	NA
Kenya				
Kakuma	− 0.53	− 0.57	− 0.49	0.000
Kalobeyei	− 0.47	− 0.52	− 0.43	0.000
South Sudan				
Ajoung Thok	−0.28	− 0.32	− 0.23	0.000
Pamir	−0.34	−0.39	− 0.29	0.000
Uganda				
Kyangwali	NA	NA	NA	NA
Palabek	−0.49	−0.53	−0.46	0.000
Zimbabwe				
Tongogara	−0.64	−0.69	− 0.58	0.000
Household size				
1 to 3 members	0.00	0.00	0.00	NA
4 to 6 members	0.11	0.10	0.13	0.000
7 or more members	0.15	0.14	0.17	0.000
Member/s with disability or who are elderly				
No	0.00	0.00	0.00	NA
Yes	−0.06	−0.07	−0.05	0.000

Source: Table created from the Standardized Water, Sanitation and Hygiene, Knowledge, Attitude and Practice Surveys, UNHCR, 2019

^a^Kyangwali refugee camp not included in the multivariate linear regression

## Discussion

Important insights regarding WASH access in refugee camps can be gleaned when comparing them to rates seen in each camp’s host country (Supplementary Table 2). Following the definitions used by the national statistics offices of each country, all the refugee camps and settlements included in this study, would be classified as an urban area and therefore comparable to urban data from JMP. However, in many cases these camps or settlements are in rural areas and were initially established in areas devoid of any public services.

### Water supply

We found high levels of access to improved water in refugee camps: on average 95% of households surveyed accessed an improved water source. Two camps had low household access to improved water sources: Tongogara, Zimbabwe (57%) and Kyangwali, Uganda (67%). These camps are the oldest sites, established in 1984 and 1960, and have been undergoing upgrades to their water supply systems since the surveys in 2019. The upgrades in Tongogara camp could be a result of damages following Cyclone Idai which hit in early 2019.

Water access in refugee camps was largely comparable to water access for urban households in host countries. Since urban coverage is nearly always higher than in rural areas, this meant that refugee access to improved water was higher than the national JMP estimates. Zimbabwe was one exception; 98% of urban households access improved sources and only 57% do in the Tongogara RC, an interesting deviation that may deserve additional study.

We found low water supply rates, corresponding to studies in other refugee camps [52]. The Ajoung Thok camp in South Sudan was the only observed locus with more than 50% households accessing 20 L/p/d. Bangladesh camps had the lowest water supply access rates. Of the 12 sites with 50% or less meeting this minimum water supply target (15 L/p/d), ten were from Bangladesh. The other two sites, Kalobeyei and Kakuma, are in Turkana County in Northwestern Kenya which was experiencing extreme droughts in 2019. It is important to note that Bangladeshi camps were in an emergency and this may explain why they have very few households accessing 20 L/p/d, as this is a post-emergency target [1].

### Sanitation

Overall, there were high levels of shared sanitation in camps. Studies in refugee populations found that shared sanitation has been related to infectious diseases and in recent years also to the spread of COVID-19 [53, 54]. The camps that listed higher open defecation rates (Kalobeyei in Kenya, and Palabek and Kyangwali in Uganda) have lower population density and are surrounded by open undeveloped land which may be more conducive to this practice. Open defecation was lowest in Bangladesh. This may be due to the generally high latrine coverage and high population density which may inhibit open defecation. In addition, in the Bangladeshi camps nearly all of sanitation facilities observed were improved. This reflects a major investment in sanitation infrastructure made by humanitarian actors, partially in response to the high risk of a cholera or other diarrheal disease outbreak- as Bangladesh is a cholera endemic country and the refugee sites have extremely high population density.

Bangladesh camps had much lower access to basic sanitation compared to other sites, and to urban country-level data, as shared facilities are the norm in the Bangladesh camps due to space constraints. In Uganda, refugee camps had similar WASH access to urban areas throughout the country which in Kenya, South Sudan, and Zimbabwe, refugee camps had better access to basic sanitation facilities when compared to urban areas of their host countries. This may be due to the age of these camps and the tendency for UNHCR and partners to support the move towards household level (i.e., non-shared) sanitation services through subsidies and distributions of concrete or plastic latrine slabs to households. Such subsidies are usually avoided in sanitation programming (i.e., that which would impact the host country urban households).

Most of the refugee sites had low rates of basic sanitation and sanitation privacy coverage. A door and lock in sanitation facilities could benefit women and girls and could deter GBV inside the sanitation facilities, but may not decrease the risks related to walking to these facilities when they are far from the household, and especially at night [17, 18]. Qualitative research on sanitation-related GBV highlights a range of vulnerabilities faced by refugee or internally displaced women and girls who are forced to openly defecate or walk to shared sanitation facilities [15]. Communal shared latrine are less safe for women and access to these types of facilities put women at risk of sexual violence and harassment [18]. Our research provides some of the first quantitative evidence of WASH access in refugee camps/settlements, with a specific focus on women, menstrual health, and their specific WASH needs. Collecting GBV data in the future implementation of KAP surveys in refugee populations could provide relevant information to help protect women and girls.

### Hygiene

According to the JMP the presence of a handwashing facility with soap and water is a priority indicator for the global monitoring of hygiene [11]. In the refugee camps and settlements studied, however, only 24% of all households surveyed have access to these facilities. Previous studies have reported low handwashing practice rates carried out in refugee camps [55, 56]. This is an important area to improve in refugee camps, as handwashing with soap is critical for general hygiene practice, menstrual health [19, 24], and to reduce the spread of COVID-19 (SARS-CoV-2) [57, 58].

The only camps that had over 50% of households with basic hand hygiene facilities were in Bangladesh. The high population density and the concerns related to cholera, may have improved soap distributions in the humanitarian response in Bangladesh. For the older camps, like Tongogara, Zimbabwe (1984), Kyangwali, Uganda (1960), and Kakuma, Kenya (1992), lower levels of soap access may be the result of per capita expenditure on WASH decreasing in camps hosting protracted displacements. Basic hand hygiene access in camps and settlements was lower than urban level data in host countries except for South Sudan where no JMP data was available.

Households with women of reproductive age were much more likely to have a bathing structure. Women of reproductive age may be less willing to bath in community facilities and more interested in constructing bathing facilities in their own homes, based on their vulnerability to harassment in shared facilities [18]. In camps in Bangladesh, it is common to have “wet areas” constructed in dwellings which discharge into a network of surface water drains where household members bathe. While this has benefits in terms of privacy, safety, and reduced risk to GBV for women, there are negative health implications related to inadequate grey water management. Preliminary research in Bangladesh has shown that the effluent from the self-made bathing structures includes a considerable number of pathogens (e.g. fecal coliform levels) [59].

Access to menstrual health materials, bathing and private sanitation facilities, improved water, and toilet paper need to be improved in refugee camps and settlements studied here. In Bangladesh and South Sudan sites, access to menstrual health materials was quite low and need to be improved. However, access to menstrual health materials and other WASH resources is just one part of menstrual health, which also includes access to health information, identification and mitigation of stigma and psychological distress related to menstruation [19]. Future studies should take into consideration the data required for a comprehensive assessment of menstrual health.

### Solid waste

Looking at site-level data, 14 of 21 sites have less than two-thirds of households with access to solid waste facilities. Of those, ten were camps in Bangladesh. This is unsurprising given that the emergency in Bangladesh is the most recent of the sites studied and often solid waste services are the last to be developed. The first draft operational plan for the Solid Waste Management Strategy covering the Bangladesh refugee camps, was not finalized until July 2019, the same month that the household surveys were completed there.

### WASH inequality

WASH access is a priority for human rights and the realization of SDG 6 and 10, especially amongst vulnerable populations. Our study identified several WASH access needs in refugee camps and settlements, which require further attention. We found specific WASH access vulnerabilities based on gender, household size, disabilities and elderly status in refugee camps and settlements.

The results presented here concur with prior studies, and suggest that indices can serve as useful tools to study WASH access in refugee contexts [39–43]. The results show that inequity can be hidden when research is not disaggregated to look at disadvantaged groups/strata.

In future studies, this index could be fortified with the inclusion of additional WASH indicators related to the specific needs of females and other vulnerable groups. Such indicators could include: separate-sex latrines with doors and locks, disability-friendly facilities, and distance to sanitation facility [60].

The camps and settlements included in this study represent a range of populations (6836 people to 153,593 people) and population densities (40,000 people per km [2] in Kutapalong, Bangladesh to 14,600 people per km [2] in Pamir, South Sudab) [61]. In fact, even when compared to metropoles like New York City or Tokyo, the Bangladesh camps are some of the densest human settlements on the planet. The sites in this study also varied in age: the oldest camp, Kwangwali, Uganda is over 60 years old and the newest camps in Bangladesh are less than 4 years old. Some of the older camps continue to receive waves of refugees (e.g., Kutapalong and Nayapara, Bangladesh, Kyangwali, Uganda, and Kakuma, Kenya), while others have had relatively stable populations (e.g., Tongogara). Humanitarian expenditures and prioritization of WASH services can vary across sites within a country and across countries. The maturity of markets and availability of goods and supplies also impact the ease of access to WASH infrastructure and services.

The enabling environment of the host country can impact WASH service availability, which includes policies, legal framework, and governance structures related to refugees broadly and to the WASH sector specifically. Although humanitarian actors play an important role in refugee response and WASH service provision, it is the government that has the ultimate authority within each sovereign territory. Progressive governments can create an enabling environment that facilitates increased WASH services and increases refugee self-reliance. Such policies may include providing refugees with the right to work and earn an income, the right to establish businesses or own property, and the right to access public services (e.g., pay for household connections to the water, sewer, and electricity networks).

The enabling environment for WASH services within South Sudan and Zimbabwe, when compared to other countries, is less favorable. Due to political instability and violence, both Zimbabwe and South Sudan have produced more refugees and migrants in the past 5 years than they are hosting. The South Sudanese situation is entering its sixth year and has resulted in the displacement of over 2.2 million refugees from South Sudan who are hosted in neighboring countries like Sudan, Uganda, Ethiopia, Kenya, and the Democratic Republic of the Congo. A further 1.8 million people are displaced internally in South Sudan. Zimbabwe hosts a comparatively small number of refugees (just over 20,000), as compared to the other countries included in this study, which have from 300,000 (South Sudan) to 1.4 million (Uganda) refugees [62]. Since the political destabilization in 2017, many Zimbabweans have left to neighboring countries in search of economic opportunities [63].

### Limitations

There are several limitations to our findings. The data did not include the age of the individual household members. It was therefore impossible to investigate the impact of age distribution of household members on WASH access. Future studies should include household roster information to enable disaggregation by age and gender. UNHCR carries out other household surveys that track attributes of individual household members. Further, this household-level approach may not fully capture the WASH needs among the most vulnerable members of each household [34]. One example of the importance of analytical level can be seen in a study of WASH access in Nepal [34]. The authors compared individual- and household-level WASH access between people with and without disabilities [34]. No significant differences were found of WASH access at household-level; yet, at the individual-level, people with disabilities experienced significantly greater difficulties accessing WASH [34]. Therefore, although the differences found in WASH access among refugee camps by disability or elderly status in this study were statistically significant, the inequity could potentially be a greater if the analysis were conducted at the individual level. Moreover, these two characteristics could have different gaps if we could study them separately, and with a subclassification of type of disability (e.g., motor disability).

Additionally, sexual and reproductive health, including menstrual health, are sensitive topics in some contexts, and can cause embarrassment and shame, which can introduce bias to questions related to access to menstrual information and products [21]. Although, a small proportion of women (7%) did refuse consent to answer questions related to menstrual health, some specific camps/settlements had greater missing values on the questions on this topic. These camps are Tongogara, Zimbabwe (missing data: 38%), and Kutupalong RC, Bangladesh (missing data: 31%). This could be related to social stigma and lack of trust, or privacy concerns. In addition, and most importantly, the questions on menstrual health were only asked to women respondents and by female enumerators. Therefore, if a woman of reproductive age was not present during the interview, or if the enumerator was not a woman, the questions for this module were not asked, contributing to missing data [45].

There are also insufficient data available in the datasets to be able to determine safely managed sanitation by the JMP definition. However, this will be possible in future UNHCR surveys with the updated questionnaire [46, 64].

Of note, our descriptive and logistic regression-based approach is only one way to quantify social inequities. Other approaches, such as inequity metrics like the slope of inequality index or the concentration index of health inequalities (for continuous outcomes) could be employed in future studies [65].

## Conclusion

Large inequalities in WASH access indicators were identified across refugee sites in Bangladesh, Kenya, South Sudan, Uganda, and Zimbabwe. We found high levels of access to improved water across most of the refugee camps and settlements studied. Improvements are needed in access to basic hygiene and sanitation, sanitation privacy, waste disposal, menstrual health materials, toilet paper, and water for sanitation, across the refugee sites studied.

The Female WASH Access Index piloted here is a useful tool to summarize WASH access in households with women of reproductive age in refugee contexts, highlighting important disparities and areas where resources are needed to improve the health and wellbeing of vulnerable members of society. The index pointed to lower WASH access in households with a member with a disability or who are elderly, households without at least one woman of reproductive age, and households with fewer than four members.

The results presented in this article highlight specific WASH needs in refugee camps and settlements that require specific attention. Women of reproductive age in the explored sites had several unmet needs related to WASH and menstrual health. Disability and elderly status further limited WASH access in the camps studied. Improved monitoring and evaluation of WASH access in these settings is needed at the individual level and on menstrual health. These results suggest tailored decision-making as well as humanitarian and political actions are needed to improve WASH access in refugee camps and settlements.

## Supplementary Information

**Additional file 1.**

## Data Availability

The source data were provided by UNHCR Microdata Library in accordance with the citation requirement provided with the dataset. UNHCR does not warrant in any way the accuracy of the data or information reproduced from Microdata Library and may not be held liable for any loss caused by reliance on the accuracy or reliability thereof [44].
