# Correction: Social and geographic inequalities in water, sanitation and hygiene access in 21 refugee camps and settlements in Bangladesh, Kenya, Uganda, South Sudan, and Zimbabwe

**DOI:** 10.1186/s12939-022-01671-y

**Published:** 2022-05-10

**Authors:** Alhelí Calderón-Villarreal, Ryan Schweitzer, Georgia Kayser

**Affiliations:** 1 Department of Family Medicine and Public Health, University of California, San Diego (UCSD), San Diego, California USA; 2 School of Public Health, San Diego State University (SDSU), San Diego, California USA; 3 Former United Nations Refugee Agency (UNHCR) WASH Officer, Geneva, Switzerland; 4 Division of Global Health, Herbert Wertheim School of Public Health and Human Longevity, UCSD, San Diego, California USA

**Correction to: Int J Equity Health 21, 27 (2022)**

**https://doi.org/10.1186/s12939-022-01626-3**

After publication of this article [1], the authors reported that the affiliation details for Ryan Schweitzer were incorrectly given as ‘Division of Global Health, Herbert Wertheim School of Public Health and Human Longevity, UCSD, San Diego, California, USA’, but should have been ‘Former United Nations Refugee Agency (UNHCR) WASH Officer, Geneva, Switzerland’, and the affiliation details for Georgia Kayser were incorrectly given as ‘Former United Nations Refugee Agency (UNHCR) WASH Officer, Geneva, Switzerland’. but should have been ‘Division of Global Health, Herbert Wertheim School of Public Health and Human Longevity, UCSD, San Diego, California, USA’.

The original article [1] has been updated.
