# Supplementary material for: Social and geographic inequalities in water, sanitation and hygiene access in 21 refugee camps and settlements in Bangladesh, Kenya, Uganda, South Sudan, and Zimbabwe
Source: Int J Equity Health. 2022 Feb 19;21:27. doi: 10.1186/s12939-022-01626-3 (PMC8857872; doi:10.1186/s12939-022-01626-3)
Supplement: Supplementary file 1 — Additional file 1. [file 12939_2022_1626_MOESM1_ESM.zip › Supplements.docx]

**Supplements**

**Table 1.** Missing values of WASH access indicators in 21 refugee camps in 2019

**Table 2.** Comparison of household WASH access at urban level in host countries, JMP and WASH-KAP 2019

**Supplementary Figures and Captions**

**Figure 1.** Percentage of Household by WASH Access Indicators in 21 Refugee Camps by Country in 2019

This heat map illustrates the percentage of households with WASH access according to 21 refugee camps in Bangladesh, Kenya, South Sudan, Uganda and Zimbabwe in 2019. On the y axis, are the six WASH indicators utilized in the female WASH access index, and on the x axis the name of refugee camps followed by the abbreviation of the settlement country. The lighter the color in the box, the lower the percentage of households with WASH access in the corresponding refugee camp. This figure was created from the Standardized WASH KAP Surveys, UNHCR, 2019.

**
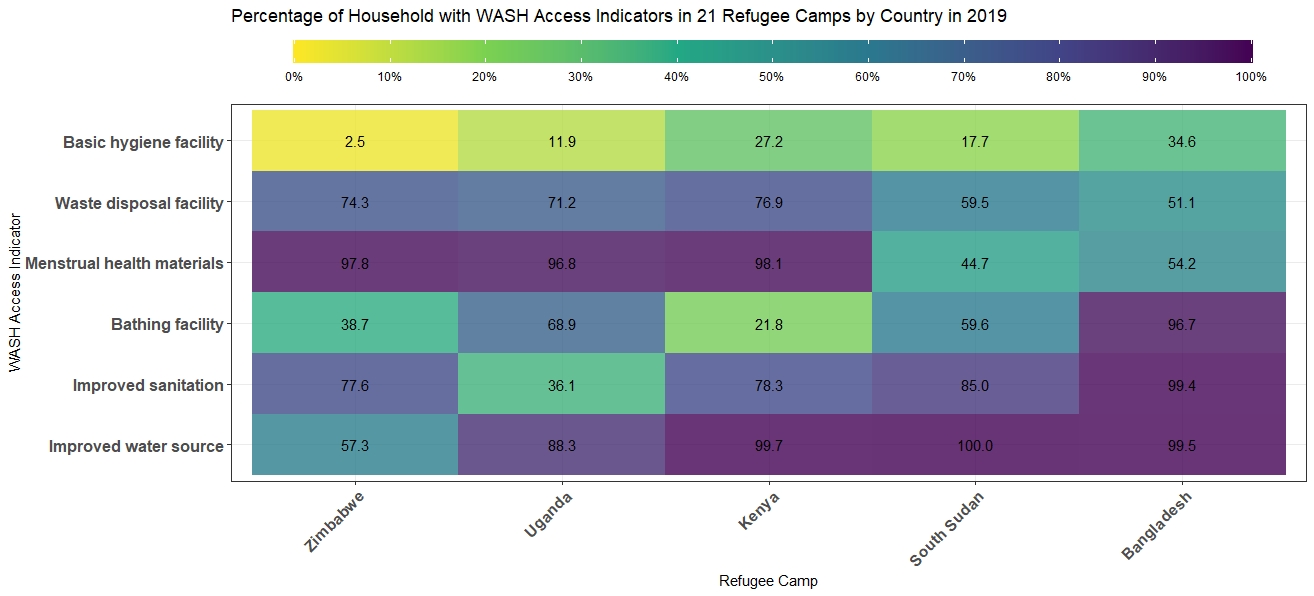
**

**Figure 2.** Female WASH Access Index in 21 Refugee Camps by Country in 2019

This bar graph illustrates the Female WASH Access Index according to 21 refugee camps in Bangladesh, Kenya, South Sudan, Uganda and Zimbabwe in 2019. On the y axis, is the Female WASH Access Index value, and on the x axis the name of refugee camps. Each settlement country is indicated by a different color. This figure was created from the Standardized WASH KAP Surveys, UNHCR, 2019.


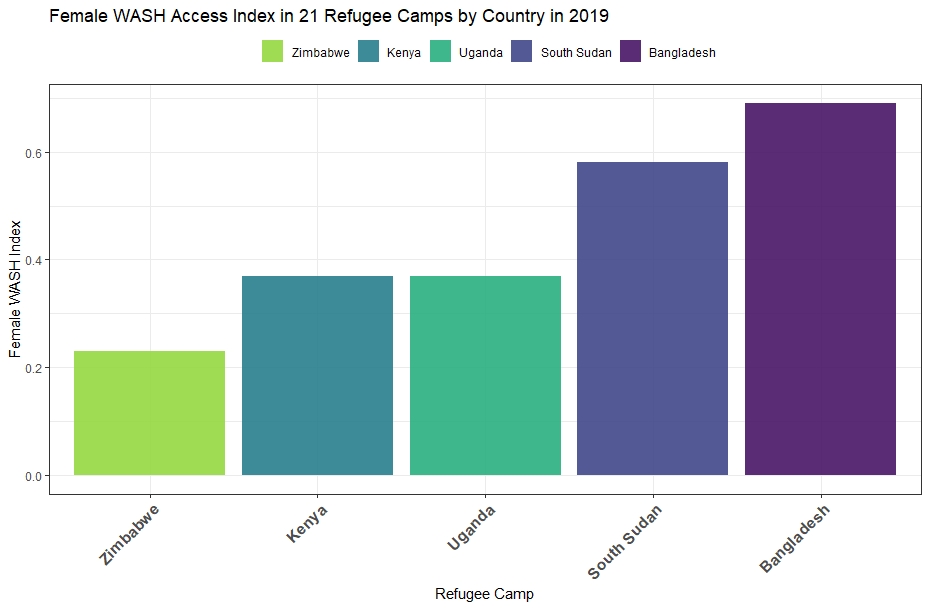


**Questionnaire.** The below question represents only the analysed sections and questions of the Global WASH KAP Standardized Survey, UNHCR.

*General Information and Demographics*

| **How many people live and slept in this house last night?** _____ people |
| --- |
| **Are there any persons with disabilities and / or elders (60+) in this household?**  🞅 Yes  🞅 No |

*Water Collection and Storage*

| **What is the principal source of domestic drinking water for members of your household?** (*Check one but do not prompt with responses. Consider water for drinking, cooking, bathing, personal hygiene, laundry and cleaning only – NOT for non-domestic use.*)  🞅 Public tap/standpipe  🞅 Handpumps/boreholes  🞅 Unprotected hand-dug well  🞅 Water seller/kiosks  🞅 Piped connection to house (or neighbour’s house)  🞅 Surface water (lake, pond, dam, river)  🞅 Protected spring  🞅 Unprotected spring  🞅 Rainwater collection  🞅 Bottled water, water sachets  🞅 Tanker truck  🞅 Other (please specify): _____________________  🞅 Don’t know |
| --- |
| **Which containers were used to collect drinking water yesterday? This includes all water collected morning, afternoon, and evening** (*Complete box below)*   \| Type and size of container \| # of times it was filled that day \| \| --- \| --- \| \| Example: 10 Liters Jerry can 1 \| 2 times \| \|  \|  \| \|  \|  \| |

*Hygiene*

| **Please show me the soap you have in the household.** *(Check one)*  🞅 Presented within one minute  🞅 Not presented within one minute |
| --- |
| **Is there a specific hand washing device/station in your house where your household washes their hands?** *(Check one)*  🞅 Yes  🞅 No |
| **Is there water in the hand washing device/station?** *(Check one)*  🞅 Yes  🞅 No |
| **Is there soap in the area of the hand washing device/station?** *(Check one)*  🞅 Yes  🞅 No |

*Sanitation*

| **Where do you and your household members (excluding children under 5) usually go to defecate?** *(Is considered communal – or shared – a latrine used by more than one household. Check one*)  🞅 Household latrine  🞅 Communal latrine  🞅 Open defecation  🞅 Plastic bag  🞅 Bucket Toilet  🞅 Other  🞅 Don’t know |
| --- |
| **Observe type of slab present** *(Verify whether in its current state – slab and superstructure – the latrine is usable.* *Check one*)  🞅 Wood  🞅 Logs  🞅 Plastic  🞅 Concrete  🞅 Other (please specify):____________________  🞅 None  🞅 Don’t know |
| **Does this latrine provide adequate privacy for you and your household members?** *(Check one)*  🞅 Yes  🞅 No  🞅 No latrine  🞅 Don’t know |
| **Is there a handwashing station at the latrine?** *(Check one)*  🞅 Yes  🞅 No |
| **Indicate whether there is water in the handwashing station** (*Check one*)  🞅 Yes  🞅 No |
| **Is soap present at the hand washing station?** *(Check one)*  🞅 Yes  🞅 No |
| **Please show me the facility where you and your family members bathe?** *(Observe if they have a designated facility at home and check one)*  🞅 Do not have a designated bathing facility  🞅 Have a designated shower/bathing facility  🞅 Don’t know or can’t observe |
| **Where does your household dispose of domestic waste?** *(Check one)*  🞅 Household pit  🞅 Communal pit  🞅 Street bin/container for garbage collection  🞅 Designated open area  🞅 Undesignated open area  🞅 Bury it  🞅 Burn it  🞅 Other (please specify): ____________________ |

*Menstrual Hygiene (Female interviewers will be required for these questions)*

| **How many women of reproductive age (15-49 years old) are in this household? _____** |
| --- |
| ***Request to talk privately to that woman of the household*** |
| **What materials did you use during your last monthly period?** *(Check all that apply)*  ☐ Disposable pad  ☐Reusable pad  ☐ Reusable cloth  ☐ Tampon  ☐ Cotton  ☐ Menstrual cup  ☐ Layers of underwear  ☐ Nothing/bleed into clothes  ☐ Other (please specify): ___________________ |
| **Is toilet paper/cleaning water available where the women change their menstrual hygiene management products?** *(Check one)*  🞅 Yes  🞅 No |
