# Supplementary figures and images for: Social and geographic inequalities in water, sanitation and hygiene access in 21 refugee camps and settlements in Bangladesh, Kenya, Uganda, South Sudan, and Zimbabwe
Source: Int J Equity Health. 2022 Feb 19;21:27. doi: 10.1186/s12939-022-01626-3 (PMC8857872; doi:10.1186/s12939-022-01626-3)

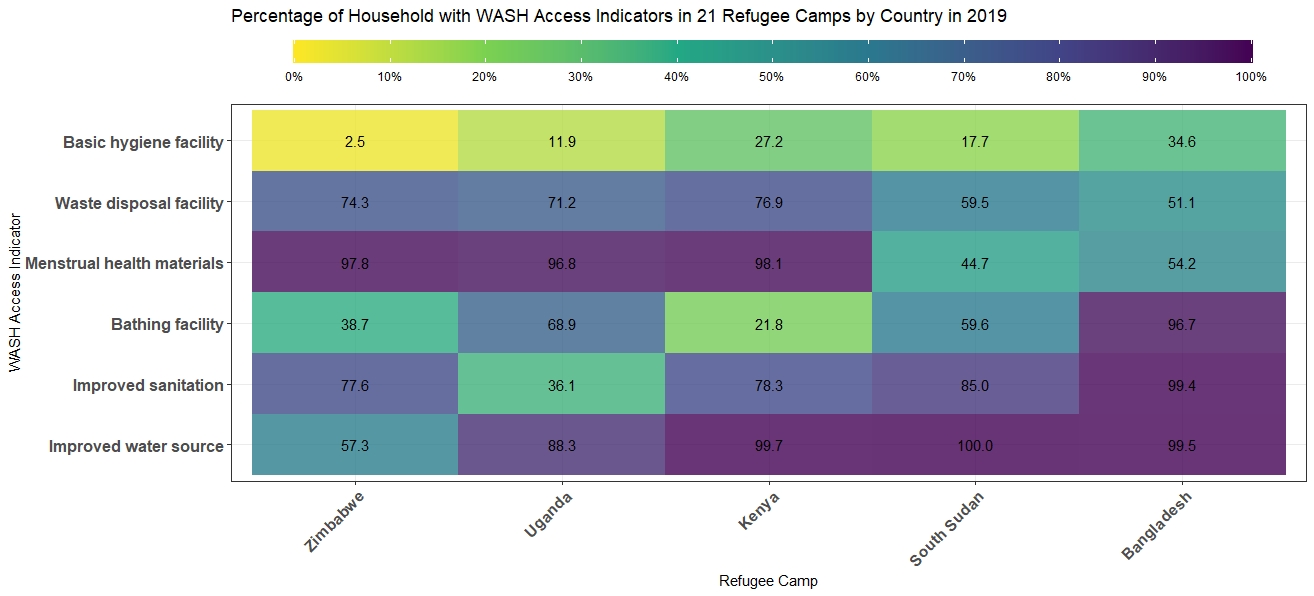

Supplement: Supplementary file 1 — Additional file 1. [file 12939_2022_1626_MOESM1_ESM.zip › Supplementary Figure 1.jpeg]

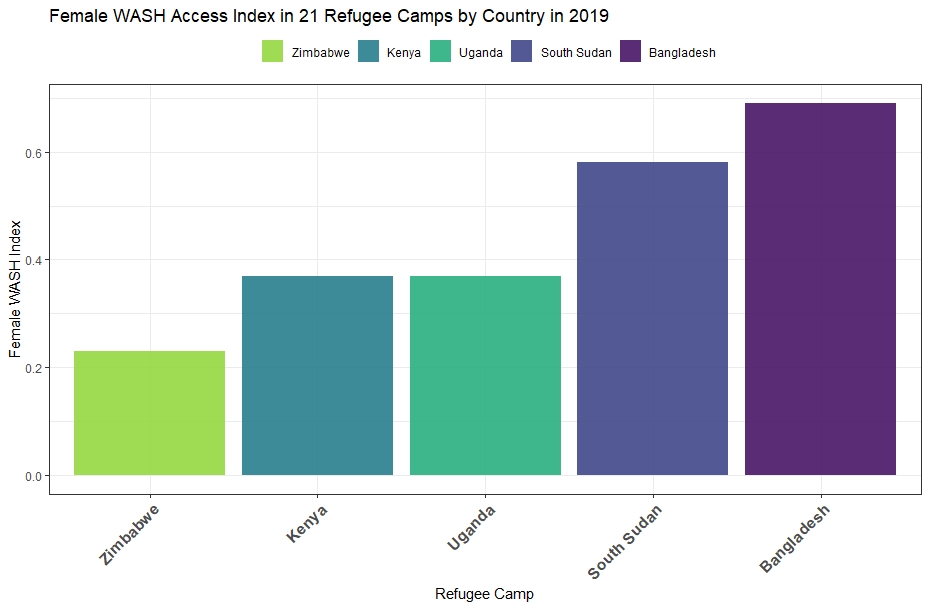

Supplement: Supplementary file 1 — Additional file 1. [file 12939_2022_1626_MOESM1_ESM.zip › Supplementary Figure 2.jpeg]
